# FEBID 3D-Nanoprinting at Low Substrate Temperatures: Pushing the Speed While Keeping the Quality

**DOI:** 10.3390/nano11061527

**Published:** 2021-06-09

**Authors:** Jakob Hinum-Wagner, David Kuhness, Gerald Kothleitner, Robert Winkler, Harald Plank

**Affiliations:** 1 Christian Doppler Laboratory for Direct–Write Fabrication of 3D Nano–Probes (DEFINE), Institute of Electron Microscopy, Graz University of Technology, Steyrergasse 17, 8010 Graz, Austria; jakob.hinum@felmi-zfe.at (J.H.-W.); david.kuhness@felmi-zfe.at (D.K.); 2 Institute of Electron Microscopy and Nanoanalysis, Graz University of Technology, Steyrergasse 17, 8010 Graz, Austria; gerald.kothleitner@felmi-zfe.at; 3 Graz Centre for Electron Microscopy, Steyrergasse 17, 8010 Graz, Austria

**Keywords:** 3D nanoprinting, direct write fabrication, additive manufacturing, focused electron beam induced deposition, 3D-nanostructures, substrate temperature, metal nanostructures, microstructure, shape fidelity

## Abstract

High-fidelity 3D printing of nanoscale objects is an increasing relevant but challenging task. Among the few fabrication techniques, focused electron beam induced deposition (FEBID) has demonstrated its high potential due to its direct-write character, nanoscale capabilities in 3D space and a very high design flexibility. A limitation, however, is the low fabrication speed, which often restricts 3D-FEBID for the fabrication of single objects. In this study, we approach that challenge by reducing the substrate temperatures with a homemade Peltier stage and investigate the effects on Pt based 3D deposits in a temperature range of 5–30 °C. The findings reveal a volume growth rate boost up to a factor of 5.6, while the shape fidelity in 3D space is maintained. From a materials point of view, the internal nanogranular composition is practically unaffected down to 10 °C, followed by a slight grain size increase for even lower temperatures. The study is complemented by a comprehensive discussion about the growth mechanism for a more general picture. The combined findings demonstrate that FEBID on low substrate temperatures is not only much faster, but practically free of drawbacks during high fidelity 3D nanofabrication.

## 1. Introduction

Three-dimensional printing of nanoscale objects is an emerging technology on the route to future applications in research and development. However, the downscaling of structural dimensions poses great challenges to fabrication methods [1]. Among the few additive manufacturing processes that allow resolution in the sub-100 nm scale, 3D nanoprinting via focused electron beam induced deposition (3D-FEBID) has demonstrated great potential. This technology uses a nanosized, focused electron beam to locally deposit material from precursor molecules temporarily adsorbed on the substrate from the gas phase. The precursor gas is locally delivered via the gas injection system (GIS) inside the vacuum chamber. By combining small lateral electron beam displacements (sub-10 nm) and long exposure times (milliseconds), the deposit lifts off from the substrate, resulting in inclined, freestanding nanowires. This 3D-FEBID technique allows 3D printing of even complex structures with nanoscale dimensions [2].

The main advantages of this nanofabrication technique are (1) the direct-write character without pre- or post-processing steps, (2) the small structural sizes down to the sub-20 nm regime [3], (3) low demands on material and substrate morphology as long as accessible by the electron beam [4], (4) the flexibility in terms of aimed shapes [5,6] and (5) the possibility to deposit various materials [7] for different functionalities ranging from electrically insulating [8]/semiconducting [9]/conductive [10] over magnetic [11] and superconducting [12] towards optical active [13]. Strong advances in recent years [2] enable the fabrication of (6) highly complex nanoarchitectures [13], with (7) improved reliability and reproducibility, without severe growth artefacts [14]. Due to such capabilities, 3D-FEBID enables novel application concepts, e.g., in scanning probe microscopy [15,16], 3D-magnetism [11,17] or as sensing devices [18,19].

While well suited as prototyping tool, 3D-FEBID has not found its broad way into industry yet. Drawbacks are the low purity due to high carbon contents in as-deposited materials for most precursors [20] and low fabrication speeds with growth rates in the range of tens of nm/s [14]. The removal of carbon contaminants from 3D-FEBID materials is discussed in detail elsewhere [13,21,22], while we address different strategies to tackle the speed issue. Under typical fabrication conditions, the amount of available precursor molecules at deposition sites limit 3D-growth (molecule limited regime [23]). To enhance the deposition rate, it is therefore necessary to establish a high precursor coverage at the actual growth front. This can be achieved by increasing the molecule flux from the gas injection nozzle towards the substrate, e.g., by increasing the crucible temperature [3], by a dedicated nozzle design [24] and by optimizing the alignment of the gas injection system [25]. However, the maximum flux is limited by precursor properties such as decomposition temperatures and pressure limits inside the vacuum chamber [20]. Another important aspect for the precursor coverage at the growth front is beam induced heating [26]. Inelastic electron scattering inside the growing wires can raise the temperature at the beam impact region for several °C [27]. At higher temperatures, more precursor molecules desorb from the wire, which gradually decreases the volume growth rate. Additionally, the increasing nanowire lengths make heat dissipation more complicated, leading to continuous temperature increase at the growth front. As a consequence, the total growth rate is further decreasing, leading to downward bending [27] of inclined segments and eventually to a collapse of 3D growth.

To avoid such unwanted growth variations along the wire length, one can apply different counter measures: (1) reducing the beam current (at the cost of volume growth rates) [3], (2) increasing the primary beam energy for less inelastic scattering events (at the cost of more circular wire cross-sectional shapes [3]), (3) introducing additional refresh times between subsequent patterning pulses [14] (at the cost of process time, or interlacing for multi-branch structures [13]) or (4) introducing a beam blur (reducing electron density and thermal resistance at the cost of minimal feature size [28]). Another approach to reduce beam induced heating effects and to increase deposition speeds at the same time is cooling the substrate. Bresin et al. demonstrated a boost of growth rate up to 4 orders of magnitude by using cryogenic substrate temperatures (Cryo-FEBID) [29]. At such temperatures (−155 °C), the precursor condenses in layers of several nanometer thickness at the substrate [30]. However, the minimal feature size and geometrical flexibility suffer and complex 3-dimensional structures cannot be realized [1]. Recently, Huth et al. analyzed the growth rates of two-dimensional pads at substrate temperatures between 5 and 24 °C for different precursors [31]. For the Pt-precursor (Me_3_CpMePt), which is also used in the present study, the deposit grew up to 6-fold higher [31]. These FEBID experiments in 2D indicate that a cooled substrate may also be suitable to increase the growth rates for 3-dimensional deposits. However, the effects of cooled substrates during 3D-nanoprinting on growth rates, shape quality and microstructure is still unclear, in particular, since the growth characteristics for flat 2D-deposits strongly differ from the growth of freestanding 3D-structures (3D-FEBID) [3].

In this work, we investigate the effects of the substrate temperature (**T_S_**) in a range of 5–30 °C on the 3D growth of multibranch PtC_X_ geometries. We first compare the heights, growth rates and wire thicknesses/widths of connected three-, four- and five-legged structures, and the growth rates after the merging zone. We then evaluate the curvature of branches fabricated at different substrate temperatures, which is highly relevant for the mechanical properties [16]. Next, we compare the microstructure of the 3D-FEBID material via high-resolution transmission electron microscopy (TEM). Finally, we discuss the variations in vertical growth rates of pillars after the merging zone, which highlight the need to consider the design of the underlying structure for accurate 3D fabrication. All results indicate that substrate cooling speeds up 3D-nanoprinting without major drawbacks on the shape quality, hence paving the way for more efficient fabrication of high-fidelity 3D nanoarchitectures.

## 2. Materials and Methods

**Figure 1:**
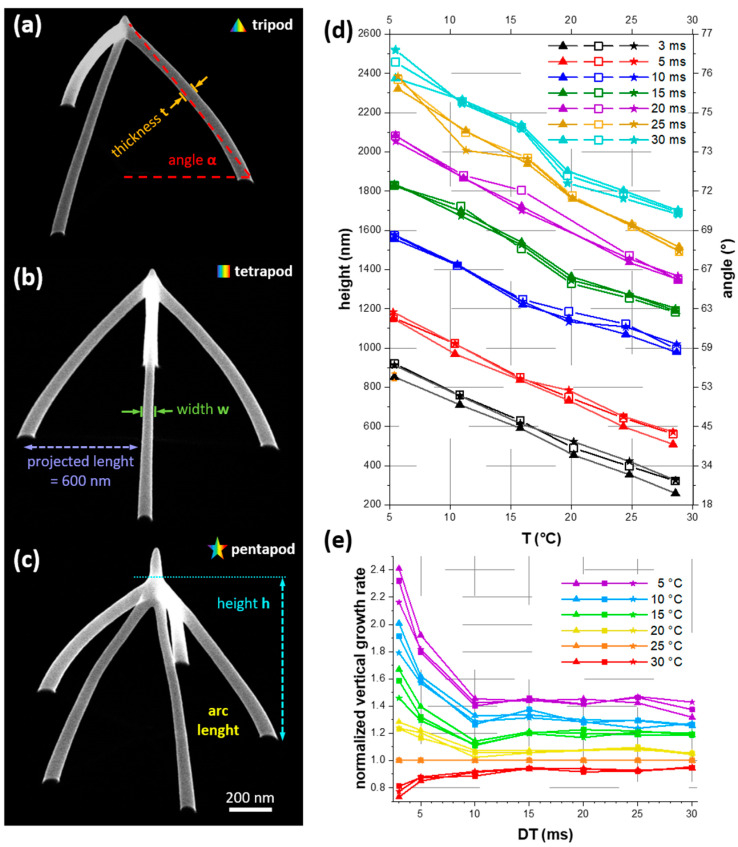
Measurements on 3D multipod nanostructures. SEM images of tripod (**a**), tetrapod (**b**) and pentapod (**c**) geometries, fabricated at 5 keV, 28 pA at constant dwell times of 3 ms and a $T_{S}$ of 5 °C. (**d**) shows the total vertical heights h as a function of the $T_{S}$ for tri-, tetra- and pentapods (indicated as triangles, rectangles and stars, respectively) fabricated with different dwell times and a projected length of 600 nm. (**e**) Vertical growth rates normalized to a $T_{S}$ of 25 °C.

3D-FEBID was performed in an SEM/FIB (Scanning Electron Microscope/Focused Ion Beam Microscope) dual beam system (Quanta 3D-FEG, FEI, Eindhoven, The Netherlands) at a primary beam energy of 5 keV and a beam current of 28 pA. For those beam parameters a strong influence of beam induced heating has been observed in a previous study [3] and a pronounced impact of substrate cooling is expected. Platinum precursor (MeCpPt^(IV)^Me_3_, CAS: 94442−22−5) is delivered via a FEI standard gas injection system [32] positioned at an angle of 52°, in a distance of 100 µm above the substrate, and in a projected radial distance of 125 µm to the deposition site. For equilibrium conditions the precursor reservoir was heated to 45 °C for at least 45 min and the gas flow was established for at least 3 min prior to any deposition experiment, which increased the chamber pressure by 7 × 10^−6^ mbar. For the experiments, multiple nanowires with a projected length of 600 nm (length in top view) were connected to a multipod structures (tripod, tetrapod and pentapod) (see Figure 1a). The inclination angles α of the branches were calculated by $\alpha \;=\tan^{-1}\;\left(h/600\right)$. Parallel writing of branches (3D-interlacing [13]) was performed at a constant step size of 1 nm and at dwell times (DT) between 3 and 30 ms, resulting in differently inclined branches. The proper writing sequence via a stream file was calculated by a homemade Matlab script. For the additional pillar growth experiments a static exposure time of 2.5 s following the multipod deposition was used.

Substrate cooling is executed via a homemade Peltier sample stage. The sample stage is driven with a QC-127-1.0-3.9M HighTech Peltier-element (QuickCool, Wuppertal, Germany). The cooling/heating power of the Peltier element was regulated by a microcontroller (TEC-1089-SV, Meerstetter Engineering, Rubigen, Switzerland). The setup was completed by an additional homemade temperature read out device based on the microcontroller AT Mega 2560 (Arduino, Monza, Italy). The substrate temperature $T_{S}$ was controlled by a PT1000 from in the vicinity of the substrate and by further NTC temperature sensors to measure the heat sink temperature. The accuracy of the temperature measurements was found to ±0.4 °C. Stable $T_{S}$ within the accuracy for at least 15 min was established in a range between 0  and 40 °C in a high vacuum atmosphere of the FIB/SEM. The maximum stable cooling rates of temperature change were 2 °C/s. Deposition experiments were conducted at approximately 5 °C, 10 °C, 15 °C, 20 °C, 25 °C and 30 °C, the exact values for each experiment are listed in Supplement 1. Due to precursor condensation and solidification of the used Pt precursor (see Supplement 2), we limited the experiments to a minimum temperature of 5 °C. After each temperature change the electron beam was carefully refocused.

For the morphological SEM study, a $1\times 1{\;\mathrm{cm}}^{2}$ silicon wafer with a 3 nm thick native oxide layer and for analysis of the microstructure via TEM 3 nm carbon films supported by lacey carbon films on 400 mesh copper grids (No 01824, TED PELLA, Redding, CA, USA) were used. Both substrates were thermally well connected to the Peltier stage. Three-dimensional-shapes were investigated via SEM images taken at 30 keV/62 pA in top view and at a 52° stage tilt. Measurements of wire width, thickness, curvature and multipod heights are performed with a self-written MATLAB image analysis script. To determine the curvature, the outer edge of a branch was interpolated by a polynomic 2nd order [16]. Error bars (omitted for the sake of clarity in graphs) were ±20 nm for height and ±3 nm for width and thickness measurements.

TEM measurements were performed with a Tecnai F20 microscope (FEI Company, Eindhoven, The Netherlands), operated at 200 keV. The sample was mounted onto a double-tilt sample-holder and measured at a tilt of 30°. Analyses were done with the software packages FIJI [33] (which is an ImageJ package [34]) and digital micrograph (Gatan Microscopy Suite, Version 3.30.2016.0, Pleasanton, CA, USA).

## 3. Results

To evaluate the influence of the $T_{S}$ on 3D-growth, we first analyzed the geometrical shape of 3D-printed nano-objects. We deposited tri-, tetra- and pentapod structures at each temperature step (5 °C, 10 °C, 15 °C, 20 °C, 25 °C and 30 °C) on silicon substrates. Figure 1a–c representatively show those three types of multipod geometries and the measurands of interest. Figure 1d displays multipod heights (left ordinate) and the corresponding inclination angles (right ordinate, calculated by $\alpha =\tan^{-1}\left(\mathrm{height}/\mathrm{projected}\;\mathrm{length}\right)$) when changing the $T_{S}$. Comparing the structure heights h reveals that for a given fabrication time, the multipods get higher at lower $T_{S}$ for all dwell times DT (see legend). As evident, the number of legs (indicated by different symbols according to a-c) is of minor importance for overall heights, since tri-, tetra- and pentapods exhibited almost identical heights for same DTs. Figure 1e shows the substrate-dependent boost in vertical growth rate normalized to the values at 25 °C (see legend). In numbers, multipods fabricated with DT>10 ms were about 50% taller compared to those at 25 °C. For low DTs (<10 ms), vertical growth was strongly enhanced up to a factor of 2.4, which in reverse means that one needs only 42 % of the process time to achieve the same object height as at 25 °C. Assuming that these trends continue, we suggest for shortest fabrication times to use (A) lowest $T_{S}$ and (B) shortest dwell times DT. For (A), the minimum temperature is determined by precursor condensation, which was found close to 0 °C for our precursor (Supplement 2). For (B), the dwell time is limited by the deflection speed of the electron beam.

**Figure 2:**
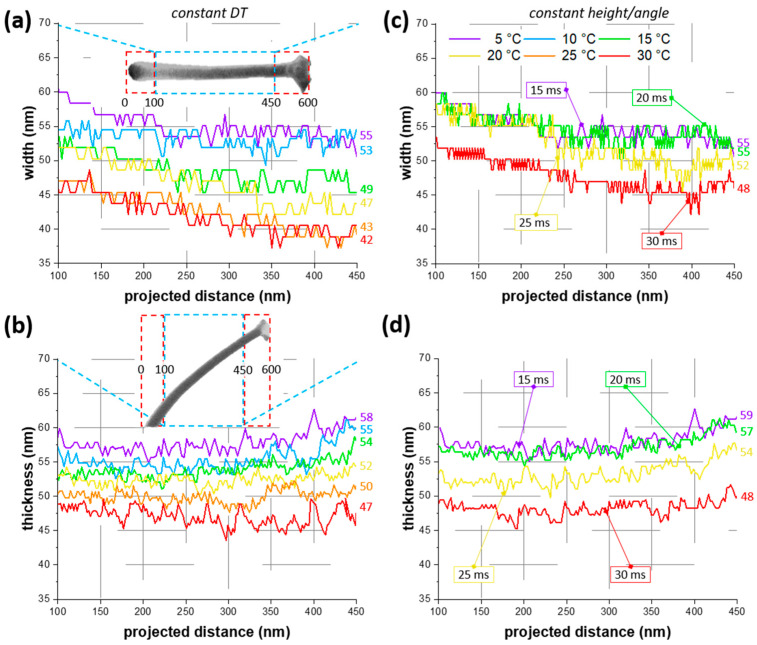
Width and thickness variations along the wires of a tripod. (**a**) Width and (**b**) thickness fabricated at a constant dwell time of 15 ms as a function of the wire length. The insets show a tripod leg in the (**a**) top and (**b**) 52° tilted view, where red boxes indicate which regions are excluded. (**c**,**d**) show the widths and thickness for tripods with nearly constant heights/angles. Values in the colored boxes specify the required dwell times for tripod fabrication. The colored numbers at the right of each graph show the mean values for thickness and width, respectively. The legend in (**c**) applies to all graphs.

High-resolution SEM imaging in top and tilted view allow to access the wire width w and wire thickness t, which both typically vary along the wire length. Figure 2 shows this evolution of width (a, c) and thickness (b, d) for tripod branches (tetra- and pentapods can be found in Supplement 3). Here, we excluded the first 100 nm and last 150 nm from the total projected length of 600 nm, as indicated in the insets in Figure 2a,b, as the lift-off region and merging zone do not represent general wire growth. Figure 2a,b shows the w- and t-evolution along the wires, fabricated at constant DTs of 15 ms. While widths were decreasing by about 10% for all dwell times, the thicknesses were widely constant along the wire. Please note that we assigned the small increase at around 450 nm projected distance to the onset of the merging zone. Aside of that on-wire variation, t and w  revealed a general increase by 30% and 18%, respectively, when lowering $T_{S}$ from 25 to 5 °C.

Figure 2a,b shows the w- and t- evolution for tripods fabricated at constant dwell times (=constant fabrication time). In this representation, however, the tripod heights were changing with the $T_{S}$ (Figure 1d). To decouple the influence from the h, we evaluated the w- and t- evolution for tripods with nearly equal heights (=inclination angle). Figure 2c,d shows the width and thickness for tripods fabricated at 10 °C, 20 °C and 30 °C using DTs of 20 ms, 25 ms and 30 ms, respectively, in comparison to the one deposited at 5 °C/15 ms (as it was used in Figure 2a,b). This constant height (or constant angle, since $\alpha \;=\tan^{-1}\left(h/600\right)$) representation also revealed the widest and thickest dimensions at low $T_{S}$. However, the longer dwell times, required at higher $T_{S}$, increased both thickness and in particular the width of the wires. Therefore, for $T_{S}$ between 5 and 20 °C widths and thicknesses were similar, in particular if we consider an uncertainty of the measurements as well. In a practical case of targeting a specific multipod height, this analysis revealed that the wire width and thickness were slightly larger at lower $T_{S}$.

**Figure 3:**
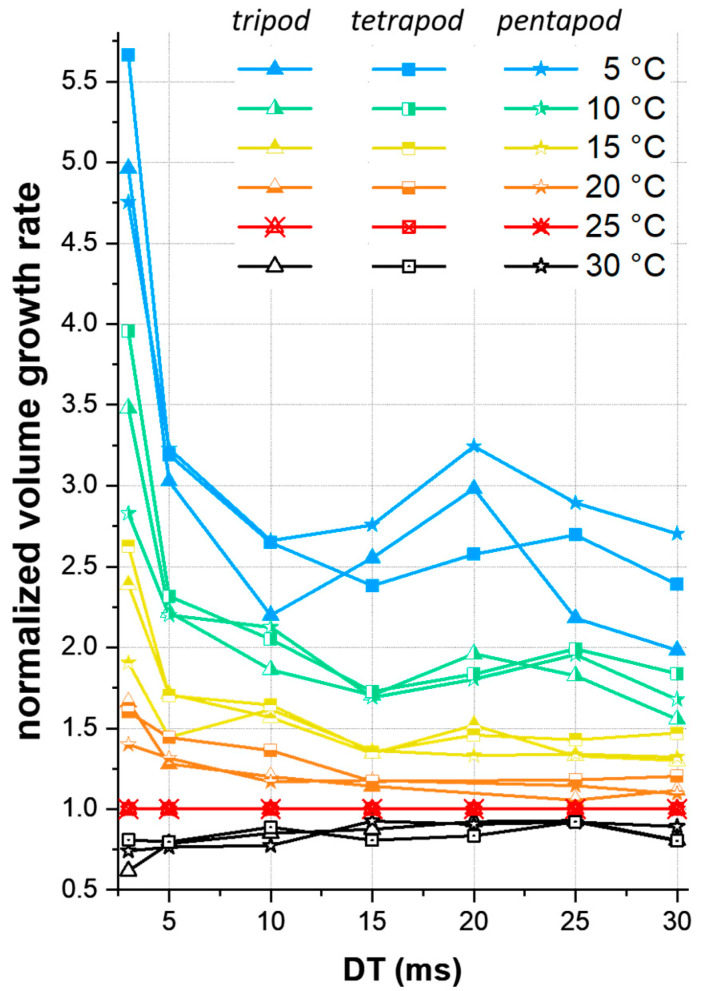
Volume growth rates of multipod geometries fabricated at different $T_{S}$, normalized to the volume growth rate at 25 °C (red dataset). The triangles, rectangles and stars indicate tri-, tetra- and pentapod structures, respectively.

Combining this enhanced growth in width and thickness (Figure 2) with the higher vertical growth rate (Figure 1b–d) consequently results in a higher volume growth rate (Vol. GR) at lower $T_{S}$. Figure 3 shows the Vol. GRs at different $T_{S}$ and for different multipod geometries normalized to 25 °C. Since w and t did not change much along the wire (Figure 2), the deposited volumes V can be approximated by V=A×L×n,  with an elliptic wire cross-section A of $A=\frac{w}{2}\times \frac{t}{2}\times \pi$, a wire length L of $L=\sqrt{h^{2}+600^{2}}$ and number of legs n [3].

Figure 3 reveals that for the same process times, up to 5.7 times more material was deposited at 5 °C compared to 25 °C. This in turn means that this low-$T_{S}$ approach drastically reduces the required process times for the same object. The volume growth boost is in particular pronounced at low DTs (<10 ms), a trend that is also observed for vertical growth rates (Figure 1e). Furthermore, Supplement 4 shows higher volume growth rates when the number of legs was increased.

**Figure 4:**
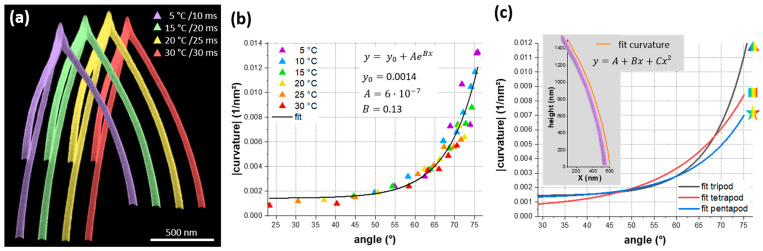
Shape quality and wire curvature. (**a**) Collage of 4 tripods with similar heights (and angles) fabricated at $T_{S}$ of 5 °C, 15 °C, 20 °C and 30 °C with dwell times of 10 ms, 20 ms, 25 ms and 30 ms, respectively, revealing high shape stability even at low temperatures. (**b**) Absolute curvature values for tripods fabricated at different $T_{S}$ (color coded) together with a common exponential fit function. (**c**) Absolute curvature fits for tri-, tetra- and pentapods. The inset representatively shows a real tripod leg together with the corresponding quadratic polynomial fit function to justify that here used modelling.

Shorter process times usually pair with a poorer shape fidelity of printed objects. We therefore evaluate on identically tall multipods, whether this cooled-substrate approach has unfavorable implications on the shape. Encouragingly, Figure 4a reveals that the tripods maintained their shape fidelity even at low $T_{S}$, which is an essential result of this study. The same also holds for tetra- and pentapods, which are shown in Supplement 5. Furthermore, we do not observe any changes in tip quality, which is of high relevance for applications such as scanning probe nanoprobes [15]. Detailed measurements of t and w (Figure 2c,d) revealed slightly larger wire dimensions at low $T_{S}$, which, however, is even advantageous in terms of the overall mechanical rigidity. In a previous study, finite element simulations have revealed a massive drop in vertical stiffness for slightly curved tetrapod geometries compared to ideally straight wires [16]. Therefore, it is necessary to evaluate how different $T_{S}$ change the wire curvatures. The red dashed line in Figure 1a clearly shows that the wires indeed deviated from a linear geometry. We approximated the wire shape by a quadratic polynomial (see inset in Figure 4c) of which the second derivative provides a measure of curvature. Figure 4b summarizes the absolute curvatures for tripods as a function of the inclination angle and reveals that the curvature is widely independent on $T_{S}$, but strongly correlates with segment angles. The solid black curve gives a common exponential fit for all tripod curvatures. The curvatures of tetra- and pentapods revealed very similar behavior (Supplement 6) and revealed slightly lower curvatures for higher number of legs as shown by the exponential fit curves for all multipods in Figure 4c.

In a first summary, all results revealed that shape fidelities were maintained over the entire temperature range studied here. The cooled-substrate approach for 3D-FEBID was therefore a convenient tool to speed up the 3D-printing process (see, e.g., Figure 4a: process time @5 °C: 18 s; @30 °C: 54 s), without major drawbacks on the shape fidelity.

**Figure 5:**
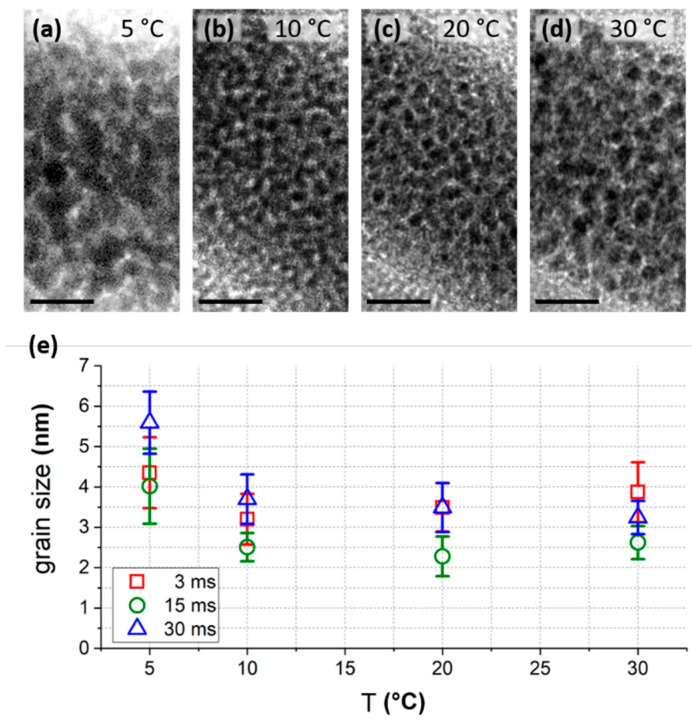
TEM microstructure analysis. In-scale comparison of TEM bright field images of tetrapod branches fabricated at (**a**) 5 °C, (**b**) 10 °C, (**c**) 20 °C and (**d**) 30 °C. Scale bars are 10 nm. (**e**) mean grain sizes (Feret-diameters) as a function of the $T_{S}$ and different dwell times, taken from tetrapod structures.

While we have shown that the overall shape is independent of $T_{S}$, the internal nano-structure could change as well. Most FEBID materials consist of nanosized metal grains embedded in a carbonaceous matrix [10,35]. The grain size is highly relevant, as it determines the physical functionality such as electrical [10], thermal [16] or mechanical properties [19,36]. We therefore conduct TEM experiments to investigate the sizes of the grains as a function of the $T_{S}$. Figure 5a–d shows four representative bright field images in the same scale, which reveal a similar microstructure for $T_{S}$ of 10–30 °C. Qualitatively, Figure 5a suggests slightly larger grains at 5 °C, which is supported by a more detailed Feret-diameter analysis of 12 tetrapods (3 DTs steps at each temperature), shown in Figure 5e. At 5 °C the mean values for the grain size ranged from 4 to 5.5 nm, while for 10 °C and higher, grain sizes between 2.2 and 4 nm were present. Note, we did not observe significant variation trends for grain sizes by using different dwell times, as evident by the different symbols in Figure 5e.

**Figure 6:**
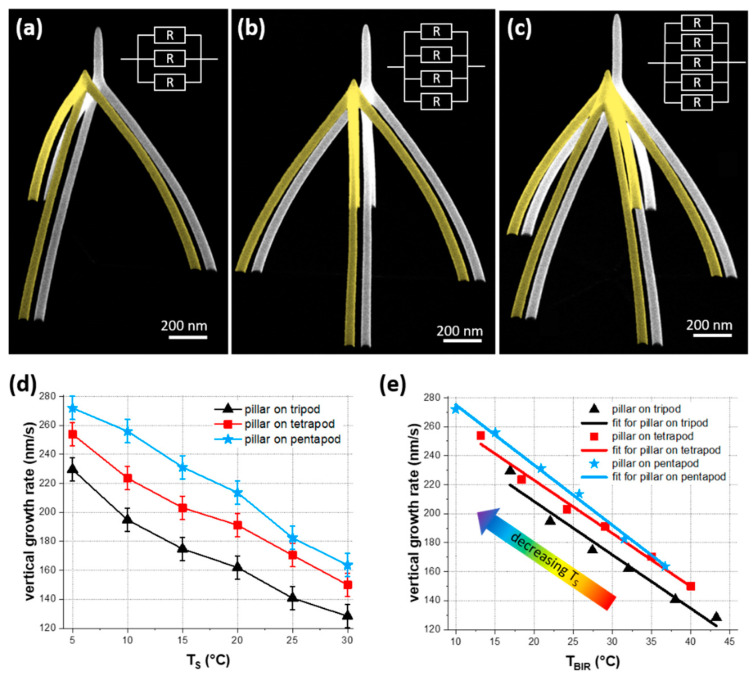
Pillar growth on multipod structures. Collage of tilted SEM images of (**a**) tripods, (**b**) tetrapods and (**c**) pentapods without (yellow) and with additional pillars (white). (**a**–**c**) were fabricated with a DT of 15 ms at a $T_{S}$ of 30 °C. The respective equivalent circuits for total thermal resistances of all structures is shown on the top right in (**a**–**c**). The vertical growth rates of the 2.5 s pillars are shown as a function of $T_{S}$ (**d**) and $T_{BIR}$ at the pillar growth front (**e**). The lines in (**e**) give linear fits of the multipods, the colored arrow indicates the trend of $T_{S}$ in the graph.

So far, we discussed multipod structures with single wires growing from the substrate and eventually merging at the tip. More complex 3D-FEBID architectures generally consists of wires that do not originate from the substrate, but have their starting point after such a merging zone [13]. To evaluate the influence of varying underlying structures, we deposited multipods with 15 ms DTs at different $T_{S}$, immediately followed by an additional pillar on top with a constant total exposure time of 2.5 s, representatively shown in Figure 6a–c for 30 °C. As reference, the according multipods from the study above are displayed in yellow. Although both series of experiments were conducted on different days, the multipods match remarkably well. This result underpins the reproducibility of FEBID-based 3D-nanoprinting. From the heights of the additional pillars, we extracted the vertical growth rate (Figure 6d), revealing the following trends: the vertical growth rate increased for (1) lower $T_{S}$ and (2) with the number of legs. During pillar deposition the electron beam generates heat at the impact region, which leads to an increased temperature in the beam impact region (BIR) at the growth front ($T_{BIR}$). Combining the total volume from the underlying multipods with the thermal resistance [26] and the measured substrate temperature, we can estimate $T_{BIR}$ (details can be found in Supplement 7). Figure 6e shows the vertical growth rate as a function of $T_{BIR}$, which reveals similar slopes of the linear fit curves for all multipod geometries, but still an offset for different leg-number, which will be discussed in detail below.

## 4. Discussion

The continuums model, which describes the FEBID process, contains several parameters that have a temperature dependence [37,38]. For the growth rate, the number of precursor molecules at the beam impact region BIR is the decisive quantity. By decreasing $T_{S}$, three mechanisms become dominant: (1) lower temperatures cause a reduced surface diffusion, which slow down the diffusive precursor replenishment [32]. Mutunga et al. have demonstrated that after artificially turning off diffusive replenishment, 3D-growth cannot be maintained [26]. (2) Lower temperatures reduce the desorption frequency of precursor molecules [26], which effectively increases the precursor residence time and by that the local coverage. Finally, (3) the sticking coefficient for impinging precursor at the surface is rises at lower $T_{S}$ [32]. Consequently, lowering $T_{S}$ reduces the number of precursor due to (1), while effects (2) and (3) boost the number of adsorbed molecules at the growth front. Which of those competing mechanisms is dominant depends on $T_{S}$ and on the precursor material [32]. For most precursors, a net decrease in growth rates has been observed upon an increase of $T_{S}$ [32], which also holds for the here used Pt-precursor, as confirmed by Huth et al. for 2-dimensional FEBID structures [31]. The results in this study also confirm an increase in growth rates for 3-dimensional FEBID structures at lower $T_{S}$ (Figure 1 and Figure 3). What might sound clear on first sight (2D→3D) is not as obvious, considering the different growth conditions for 2D compared to 3D-FEBID. In the latter, quasistatic exposure is applied, while during 2D-FEBID typical pixel pulse durations are in the range of μs to low ms. This implies major differences in pixel refresh times and beam induced heating [26]. The long and thin wires in 3D-FEBID hamper diffusive precursor replenishment in general and the efficient removal of generated Joule heat towards the heat sink—issues that are of minor relevance for 2D-deposits.

Figure 1d reveals that vertical growth rates strongly increased at lower *T_S_*, while the number of legs has a minor influence. Only for lowest DTs (3 ms and 5 ms), slightly reduced vertical growth rates for tripods compared to tetra- and pentapods are observed. Effectively, adding a further leg extended the time between two subsequent DT events at the same branch (refresh time—RT), which increased the precursor coverage at the growth front due to additional adsorption from the gas phase and diffusive replenishment. In terms of beam heating, the RT had no significant influence in this setting, since the temperature profile adjusts to the new situation (beam on/off) very quickly (on a µs time scale [26] compared to the very long stationary DTs (3–30 ms)). The almost identical heights of tri-, tetra- and pentapods in Figure 1d, fabricated at DT of 10 ms and longer, indicate that a steady-state of precursor coverage is already established for the given RT pause, with a minimum of 20 ms (3 legs with 10 ms DT). In contrast, for DTs of 3 ms we observe slightly lower tripod heights (RT=6 ms) compared to tetrapods (RT=9 ms) and pentapods (RT=12 ms). This might indicate that steady-state precursor conditions were established after a RT between 6 and 9 ms in our experiments.

Of central interest in this study is the growth boost at low $T_{S}$ compared to standard FEBID conditions (typically between 20 and 25 °C). Figure 1e reveals that multipod structures at 5 °C were about 40 % taller than at 25 °C. Remarkably is the increase at shortest DTs (3 ms in this study), where the multipods were up to 2.4 times larger than the corresponding structures at 25 °C. For highest growth efficiency, we therefore expected even higher growth rates by using shorter DTs in combination with lowest $T_{S}$. Please note that the minimum $T_{S}$ is given by precursor condensation close to 0 °C (see Supplement 2). For shortest DT pulses one has to consider technical limitations of the beam deflection system. Even stronger is the up to 5.6 times higher volume growth rate on cooled substrates (Figure 3), which stems from the enhanced spatial growth of individual segments (Figure 2a). This is again a result of a higher precursor coverage; therefore, more material is deposited in all directions and the wires get wider and thicker compared to those at higher $T_{S}$. In this context, an important observation are the still high w- and t-values compared to multipods at elevated temperatures with identical heights (Figure 2c,d). To achieve the latter structures, longer dwell- and fabrication-times are required, resulting in a higher total number of potentially dissociating electrons. For example, the process time (and therefore the number of electrons) was twice as high at 30 °C compared to 5 °C, as evident in in Figure 2c,d. Nevertheless, the mean widths and thicknesses were significantly wider at 5 °C (w=55 nm, t=59 nm) than at 30 °C (w=48 nm, t=48 nm). This indicates that for volume growth the number of precursor molecules was much more important than the number of electrons (molecule limited working regime). For a specific application, one has to decide, whether thinnest wires are essential (in this case use high $T_{S}$) or broader legs are acceptable (in this case use cooled substrates). The latter case is even more beneficial when electrical, thermal or mechanical properties are of high relevance (e.g., scanning probe concepts in scanning probe microscopy [15]).

Since temperature related effects can lead to segment-bending [26,27], a close look on segment curvatures as a function of $T_{S}$ is needed. Figure 4b reveals that curvatures increased with segment angle (or multipod height), but were widely independent on $T_{S}$. A closer look at the take-off region at the substrate revealed that the legs initially grew at steeper angles than after a few hundreds of nanometers (Figure 4a). This increased vertical growth rate at early growth stages together with the observed base broadening [22] can be explained by the much better diffusive replenishment situation close to the substrate, which acts like a much bigger precursor reservoir. With increasing segment length (distance to the substrate), growth gradually approaches a lower growth rate, leading to unwanted curvatures even at low $T_{S}$. To equalize the growth rate variations at different growth stages, one might consider an adaptive patterning velocity along the segment length [27].

When multipod legs finally merge at the tip region, the thermal resistance $R_{th\;}$ changes abruptly. While generated heat in the BIR was transported through single wires to the substrate before, there are now 2, 3 or 4 additional legs, which effectively reduces the steady-state temperature $T_{BIR}$ at the growth front. Figure 6 summarizes a dedicated experiment, where we subsequently grow an additional, vertical pillar with constant exposure time on top of tri-, tetra- and pentapods. The static exposure furthermore eliminates any RT, and by that prevents intermediate cooling and additional replenishment. Supplement 7 calculates the total thermal resistances $R_{th\;}$ of our multipods, which naturally reveals the lowest $R_{th\;}$ for pentapods fabricated at lowest $T_{S}$. Together with 3D-FEBID simulations [3,26], the temperatures at the growth front $T_{BIR}$ can be estimated, which is relevant for the subsequent pillar growth at constant beam heating rates (see Supplement 7). Consequently, higher pillar growth rates are expected on multipods fabricated at (A) lower $T_{S}$ and (B) multipods with lower $R_{th}$ (higher leg-number). Qualitatively, Figure 6d confirms these trends at first sight: according to (A), we observe increasing pillar heights by cooling the substrate, where 5 °C pillars were about 50% taller compared to 25 °C pillars. This, once again demonstrates the beneficial impact of cooled substrates on growth speed. Following argument (B), pillars got taller when the number of legs increased. Please note, this significant splitting of tri-, tetra- and pentapod data in Figure 6d was in strong contrast to the observation of widely identical heights for multipods at a given $T_{S}$ (Figure 1d), which have shown only a minor dependency on the leg number. This clearly reveals that $R_{th\;}$ of the supporting structure must be considered when aiming for accurate 3D fabrication. As all pillars in this experiment experienced the same electron dose, the differences in vertical growth rates could be attributed to a change in precursor concentration. As an increasing $T_{BIR}$ increased the desorption frequencies due to shorter mean residence time (see estimation in Supplement 8), the coverage reduction leads to shorter top pillars. Plotting the vertical growth rates (Figure 6d) as a function of $T_{BIR}$ (Figure 6e), which takes the different $R_{th}$ for multipods into account, a linear dependency is found as expected [26]. A close look on the offset between tri-, tetra- and pentapods consistently reveals a generally increasing volume growth rate for higher leg-numbers. However, there is a stronger offset between tri- and tetrapods than for tetra- to pentapods. Although more dedicated experiments are needed to get a fully comprehensive insight, the observation can be explained by a regime shift towards a more balanced situation between electrons and locally available precursor molecules for increasing leg numbers. A conceivable scenario is, that the increasing number of legs provides more paths for diffusive replenishment towards the growth front, which can shift the growth regime as mentioned above.

Aside of morphological aspects upon substrate temperature variation, the question remains, whether growth boosts and shape fidelities have to be paid with reduced material quality. For 2D-FEBID deposits, several studies have investigated compositional changes at elevated $T_{S}$ [32,39,40]. Mulders et al. have shown that some precursor materials result in higher metal content at elevated $T_{S}$ (e.g., W(CO)_6_, Co_2_(CO)_8_), while an almost temperature independent composition is found for MeCpPtMe_3_ [32]. An extrapolation of reported trends on chemical compositions for various precursors as a function of $T_{S}$ [32] down to 0 °C indicates, for which precursors we expect increased material purity when the substrate is cooled down: provided that those studies on 2D-FEBID pads are also applicable to 3D-FEBID wires we assume a lower material purity for the mentioned W and Co precursors, and only little changes for the here used the Pt precursor. For the latter Huth et al. recently reported a reduction in Pt content of 3 at.% by lowering the temperature from 24 to 5 °C [31]. The here conducted TEM analyses for 3D deposits reveal that the grain sizes in 3D were widely unchanged down to 10 °C, as summarized Figure 5. Although the grains became slightly larger at 5 °C, which can be beneficial for electric/thermal/mechanical properties, the collective findings of this study revealed that cooled substrates widely maintained the material properties of the 3D-printed wires, at least for the here used platinum precursor MeCpPtMe_3_. Consequently, the here presented cooled substrate approach for 3D-FEBID has to be evaluated for other FEBID precursors in future studies.

Finally, we want to report some practical aspects: first, a well-designed cooling stage concept is a prerequisite, since thermal drift issues can degrade the shape integrity and reproducibility. Second, $T_{S}$ is an underestimated parameter in most FEBID studies and was hardly reported in detail in the past. Most FEBID systems operate in a temperature range between 20  and 25 °C, however, even for this ΔT of 5 °C, the volume growth rate changed for about 20% (Figure 3). We therefore encourage to keep temperatures as stable as possible for reliable fabrication, and suggest to report $T_{S}$ in future studies due to the high relevance of the final results.

## 5. Conclusions

Enhanced growth rates for 3D-nanoprinting via focused electron beam induced deposition were demonstrated by using cooled substrates. This low-substrate-temperature approach significantly shortened the process times, while shape fidelities were widely maintained, aside of small increases in segment diameters. For the here used Pt precursor, we also did not find changes in material composition down to about 10 °C, while even lower temperatures of 5 °C revealed a slight grain size increase. Hence, this study demonstrated that low-substrate-temperature processing allows for reliable and accurate 3D growth without compromise but mostly advantages. However, the study also revealed that 3D designs with merging segments requires a careful look as further growth rates change due to varying thermal resistances, a detail, which becomes relevant for complex 3D, meshed architectures. By that, this work lies down the foundation for further improvement when aiming on highest spatial precision and predictability.

## Data Availability

Additional data are presented in the supplementary section and are available on request from the corresponding authors.

## Supplementary Materials

The following are available online at https://www.mdpi.com/article/10.3390/nano11061527/s1, Supplement 1: Exact fabrication temperatures; Supplement 2: Evaluation of temperature limits; Supplement 3: Thickness and width for tetra- and pentapods; Supplement 4: Absolute volume growth rates; Supplement 5: Shape fidelity of tetra- and pentapods; Supplement 6: Curvatures of tetra- and pentapods; Supplement 7: Calculation of temperatures at the growth front; Supplement 8: Calculation of mean residence times.
