# Supplementary material for: FEBID 3D-Nanoprinting at Low Substrate Temperatures: Pushing the Speed While Keeping the Quality
Source: Nanomaterials (Basel). 2021 Jun 9;11(6):1527. doi: 10.3390/nano11061527 (PMC8229455; doi:10.3390/nano11061527)
Supplement: Supplementary file 1 [file nanomaterials-11-01527-s001.zip › nanomaterials-1238720-supplementary.pdf]

Supplementary Material

FEBID 3D-Nanoprinting at Low Substrate Temperatures: Pushing the Speed While Keeping the Quality

Jakob Hinum-Wagner[1], David Kuhness[1], Gerald Kothleitner[2,3], Robert Winkler[1*] and Harald Plank[1,2,3*]

[1] Christian Doppler Laboratory for Direct–Write Fabrication of 3D Nano–Probes (DEFINE), Institute of Electron Microscopy, Graz University of Technology, Steyrergasse 17, 8010 Graz, Austria; jakob.hinum@felmi-zfe.at (J.H.-W.); david.kuhness@felmi-zfe.at (D.K.)
[2] Institute of Electron Microscopy and Nanoanalysis, Graz University of Technology, Steyrergasse 17, 8010 Graz, Austria; gerald.kothleitner@felmi-zfe.at
[3] Graz Centre for Electron Microscopy, Steyrergasse 17, 8010 Graz, Austria
* Correspondence: robert.winkler@felmi-zfe.at (R.W.); harald.plank@felmi-zfe.at (H.P.)

1. Supplement 1: Exact Fabrication Temperatures

In this study, we target substrate temperatures T_S of 5 °C, 10 °C, 15 °C, 20 °C, 25 °C and 30 °C. Depending on the cooling/heating history of each experiment (within a experimental set, dwell times (DT) were kept constant) the actual temperature stabilizes at slightly different values, which are shown in Table S1. For a concise presentation in the figures, we used the categorization in 5 °C steps thoughout the manuscript instead of the exact temperatures shown here.

Table S1. Exact substrate temperatures for each experiment. For each experimental run, the same DTs were used, whereby exact T_S were measured by a Pt1000 sensor. These values deviate slighty from the nominal temperatures. The standard deviation for the sensor data is a maximum of ± 0.4 °C.

	nominal temperatures					
**dwell time (ms)**	**5 °C (°C)**	**10 °C (°C)**	**15 °C (°C)**	**20 °C (°C)**	**25 °C (°C)**	**30 °C (°C)**
3	5.4	10.8	15.8	20.2	24.8	28.6
5	5.3	10.4	15.8	19.8	24.3	28.4
10	5.4	10.6	16.0	19.8	24.5	28.7
15	5.4	10.9	15.9	20.0	24.8	28.6
20	5.5	11.1	15.9	20.1	24.8	28.8
25	5.7	11.3	16.4	20.1	25.0	28.9
30	5.5	11.0	15.9	19.7	24.3	28.8

2. Supplement 2: Evaluation of Temperature Limits

In the following we rationalize the temperature limits for the main study via dedicated experiments. At elevated T_S a worsening of the growth rate is anticipated, we nevertheless selected a T_S of 30 °C as upper limit to demonstrate the implications on 3D-FEBID structures also above room temperature. To determine the minimum temperature, we evaluate precursor condensation effects upon very slow substrate cooling (cooling rate 0.2 °C/s). SEM images are taken every two seconds, which results in a temperature resolution of 0.4 °C. The GIS nozzle was injected (distance to substrate 100 µm) and the gas flow was active during the entire cooling process. Figure S 1 shows selected pictures of this image set. At -1.8 °C condensation of precursor is visible in front of the GIS nozzle (Figure S 1a). The condensation area increases when reducing the temperature. For Figure

S 1f, the GIS nozzle has been retracted, which reveals a circular shape of the condensate. Figure S 2 shows the subsequent evaporation process while the substrate is reheated with a temperature rate of 1 °C/s. The evaporation process is completed around 14 °C, where the condensate was brought completely to the gas phase.

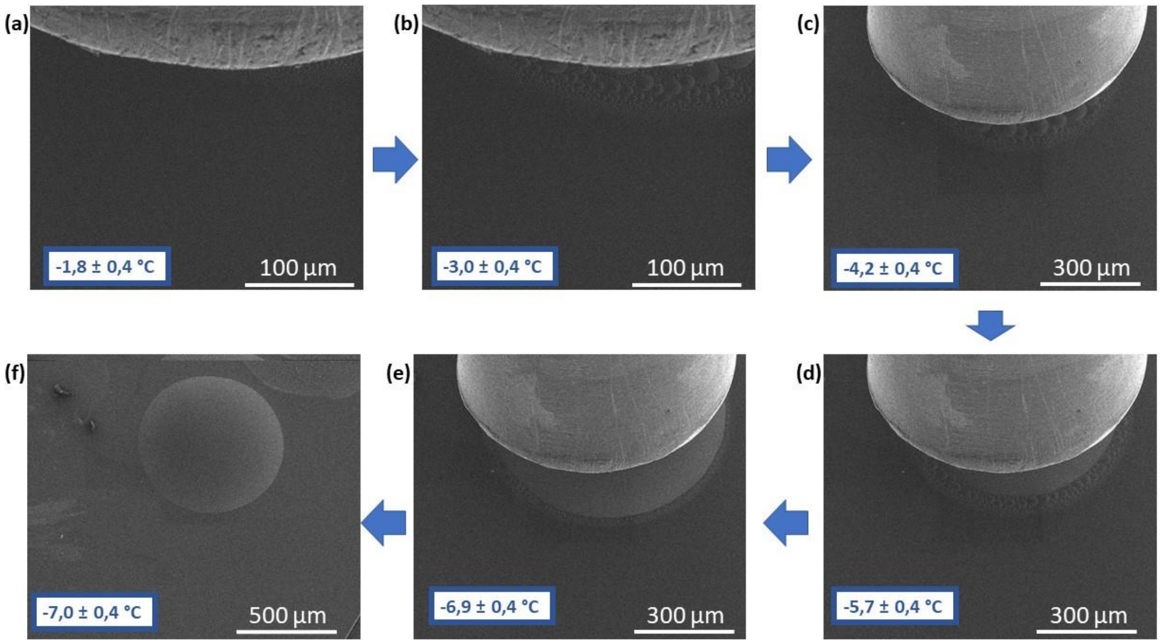

Figure S1. Precursor condensates during substrate cooling at temperatures below 0 °C **(a–f).** The gas injection nozzle is visible at the top of each SEM image in (a–e).

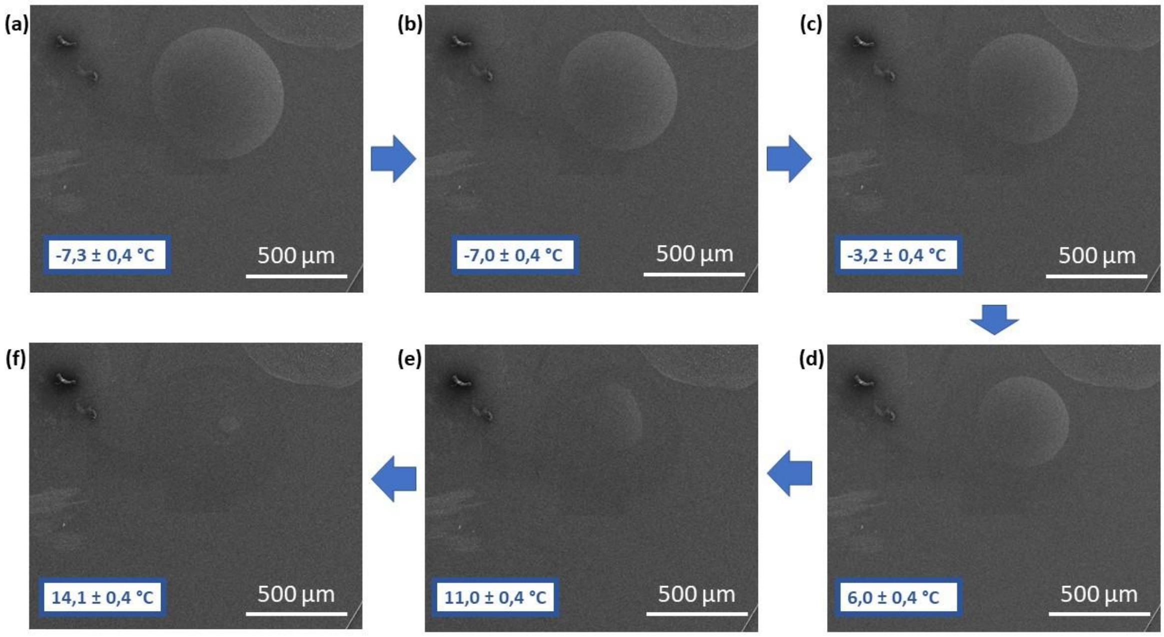

Figure S2. Evaporation of the precursor condensate when increasing the T_S from −7.3 °C to 14.1 °C.(**a**–**f**).

This experiments reveal, that for the here used Platinum precursor, T_S below 0 °C should be avoided. To assure reliable conditions we have selected 5 °C for the experiments in the main manuscript. This temperature limit minimizes the possibility of droplet

formation, avoids hysteresis effects during reheating as evident in Figure S 2, and allows to stabilize the T_S for the duration of the experiments. Please note, the Peltier cooling stage is capable for experiments below 0 °C, which might be interesting for other precursors materials in future studies.

3. Supplement 3: Thickness and Width for Tetra- and Pentapods

Figure S 3 shows widths and thickness of tetrapods and pentapods, respectively, all fabricated at constant DTs of 15 *ms*. Compared to the measurements on tripods (Figure 2a,b in the main manuscript) wire dimensions increase with the number of legs, e.g. for 5 °C: 55 nm (tripod) → 63 nm (tetrapod) → 73 nm (pentapod).

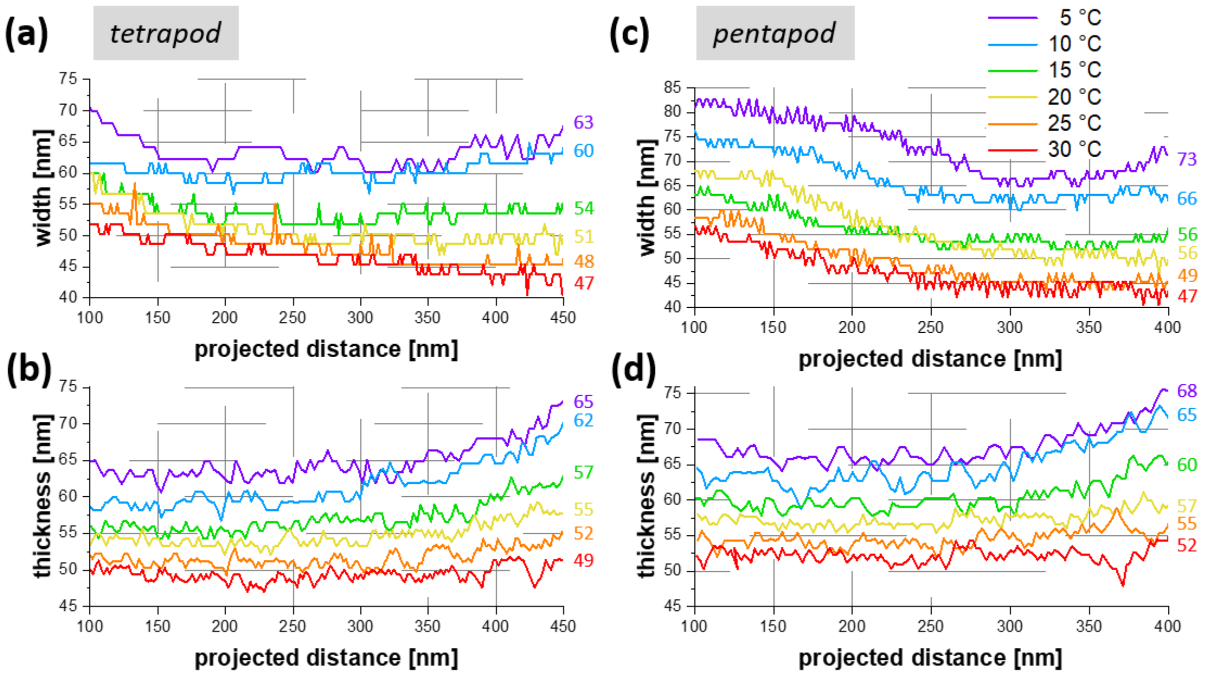

Figure S3. Width and thickness evolution for tetra- and pentapods. Width **(a,c)** and thickness **(b,d)** measurments on tetrapod and pentapod legs, respectively, fabricated with a dwell time of 15 *ms* at varying Ts. The legend in (c) applies to all graphs, the colored numbers next to each curve corresponds to the mean width/thickness in the shown region. Take-off and merging zones are excluded from the measurements.

4. Supplement 4: Absolute Volume Growth Rates

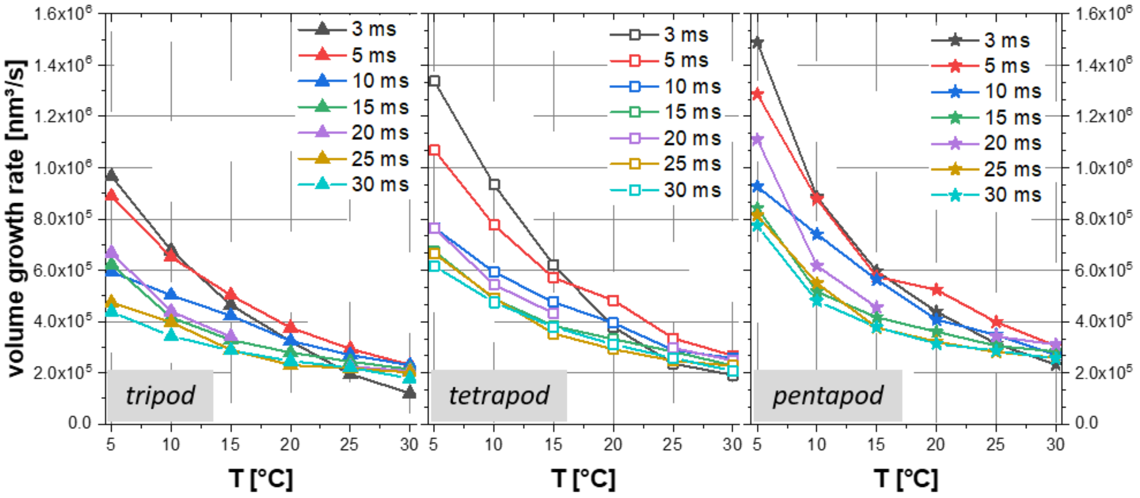

Figure S4. Volume growth rates for multipod structures as function of the T_S for different dwell time settings.

Figure S 4 shows three trends: (1) Volume growth rate (VGR) is strongly enhanced at lower T_S, (2) VGR increases with the number of legs and (3) VGR variations get larger for lower DTs. In agreement with the discussion section in the main manuscript, highest VGR (= deposition rate) is obtained at lowest T_S, low DT and longest refresh times.

5. Supplement 5: Shape Fidelity of Tetra- and Pentapods

Figure 4a in the main manuscript has demonstrated the excellent shape fidelity of tripods of similar heights fabricated at various T_S. Figure S 5 shows that this high shape quality is also valid for tetrapods and pentapods.

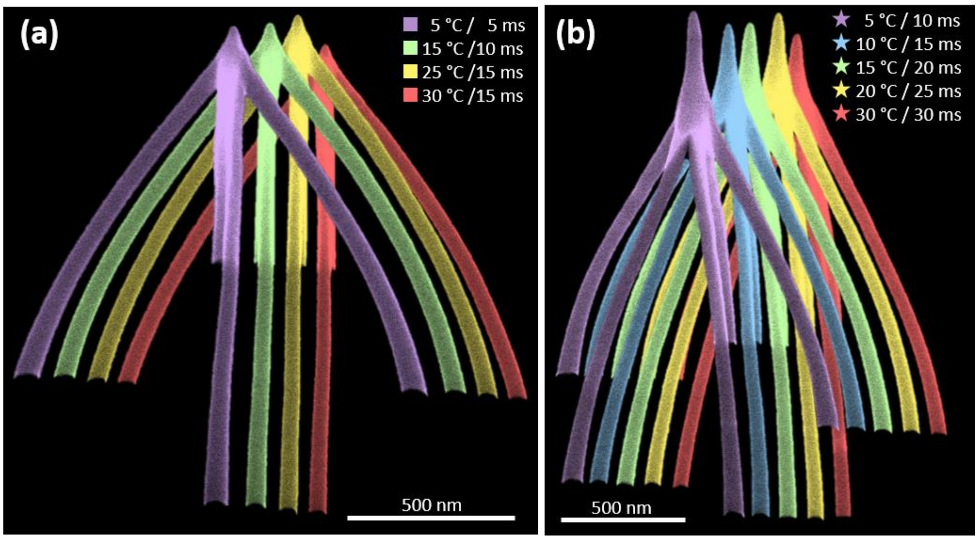

Figure S5. Tetrapods **(a)** and pentapods **(b)** of similar heights fabricated at different T_S and DT settings.

6. Supplement 6: Curvatures of Tetra- and Pentapods

Complementing the tripod data in Figure 4b in the main manuscript, Figure S 6 shows the curvatures and the corresponding fit curves for tetra- and pentapods.

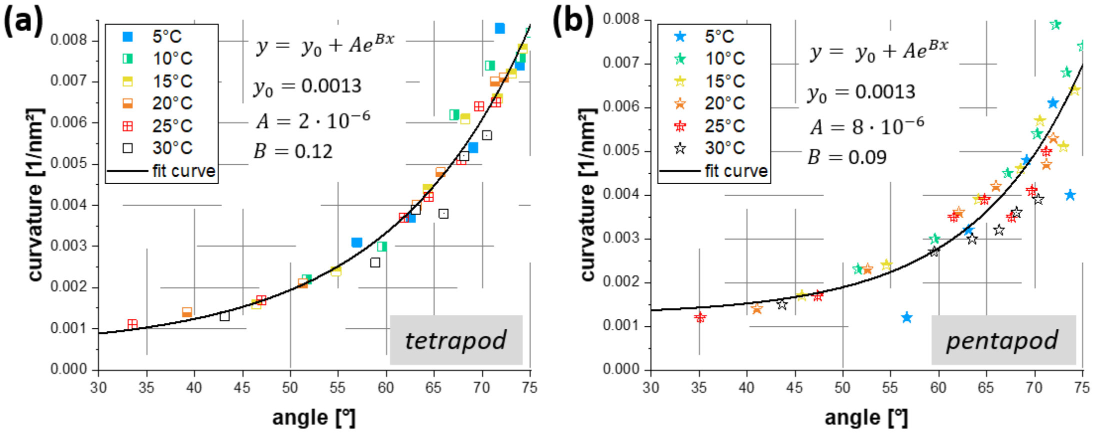

Figure S6. Curvature of tretrapod **(a)** and pentapod **(b)** branches as a function of the segment angle. The solid black lines represent an exponential fit for all temperatures down to 10 °C, the fit parameters are given in each graph.

7. Supplement 7: Calculation of Temperatures at the Growth Front

The temperature at the growth front T_{BIR} can be calculated by

$$T_{BIR} = T_S + R_{th}\frac{dQ}{dt}$$

with the thermal resistance R_{th}, and the beam heating rate $\frac{dQ}{dt}$ [1].

The thermal resistance of a single leg is given by $R_{th,leg} = \frac{L}{\lambda A}$ with thermal conductivity λ, the segment length L, and wire cross-sectional area A. Values for L and A are measured from SEM images, the cross-section area A was approximated by an ellipse. For thermal conductivity λ we used $0.24\, W/(mK)$ as reported for $5\, keV$ with similar beam currents by Fowlkes in [2]. The overall multipod thermal resistance R_{th} shown in Figure S 7a can be considered as parallel circuit of single leg resistances $R_{th,leg}$ (see Figure 6a-c top right). The higher the number of legs, the lower is the value of R_{th}. Although L increases for lower T_S for a given DT, lower values of R_{th} are found, which is caused by the larger wire widths and thicknesses at low temperatures (see Figure 2 in the main manuscript).

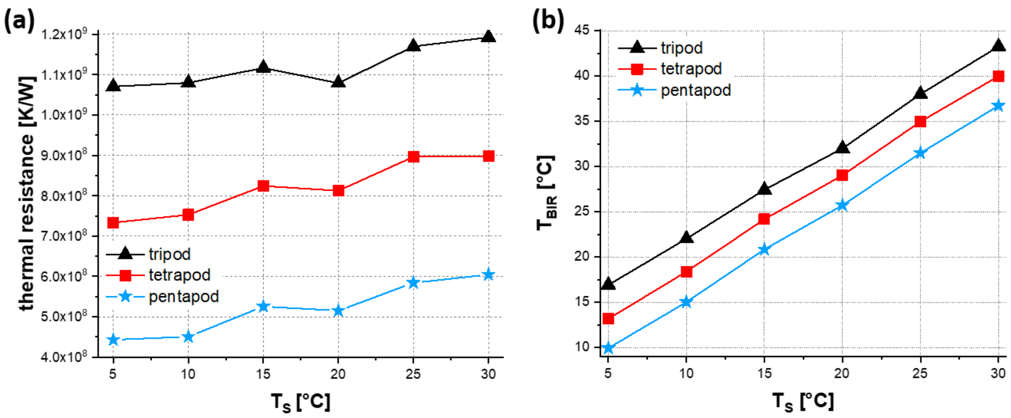

Figure S7. (a) Thermal reisistance of multipod structures and **(b)** temperatures at the pillar growth front as function of the substrate temperature T_S.

During pillar growth on the multipod, heat is generated, which is described by the beam heating rate $\frac{dQ}{dt}$. For the given beam parameters (5 keV/28 pA), we extracted a value of 1.11×10^{-8} W for $\frac{dQ}{dt}$ from simulations reported in [2], validated by complementary experiments. This allows to estimate the temperature at the growth front of a starting pillar on top of a multipod as shown in Figure S 7b. T_{BIR} is about 5 °C, 8 °C, and 12 °C higher than T_S for penta-, tetra-, and tripods, respectively. Please note, we here neglect the evolution of T_{BIR} along the vertical pillar, which is expected to be comparable small for the observed pillar heights.

8. Supplement 8: Calculation of Mean Residence Times

If T_{BIR} is known, one can calculate the mean residence time τ

$$\tau(T) = \frac{1}{k_0} e^{\frac{E_a}{k_B T}}$$

with the estimated pre-exponential attempt frequency k_0 (1×10^{13} Hz, [1]) and the activation energy for surface desorption for the $MeCpPt^{IV}Me_3$–PtC_x interface E_a ($0.62\ eV$, [1]). Figure S 8a shows the strong increase in mean precursor residence time when the T_S is reduced. Furthermore, the effects of the number of legs on the mean residence time of precursor molecules become apparent, especially at low T_S. Figure S 8b compares the vertical pillar growth rates with the respective the residence times. Please note that we only consider desorption effects here, while other temperature-dependent aspects (diffusion, sticking, dissociation) are neglected.

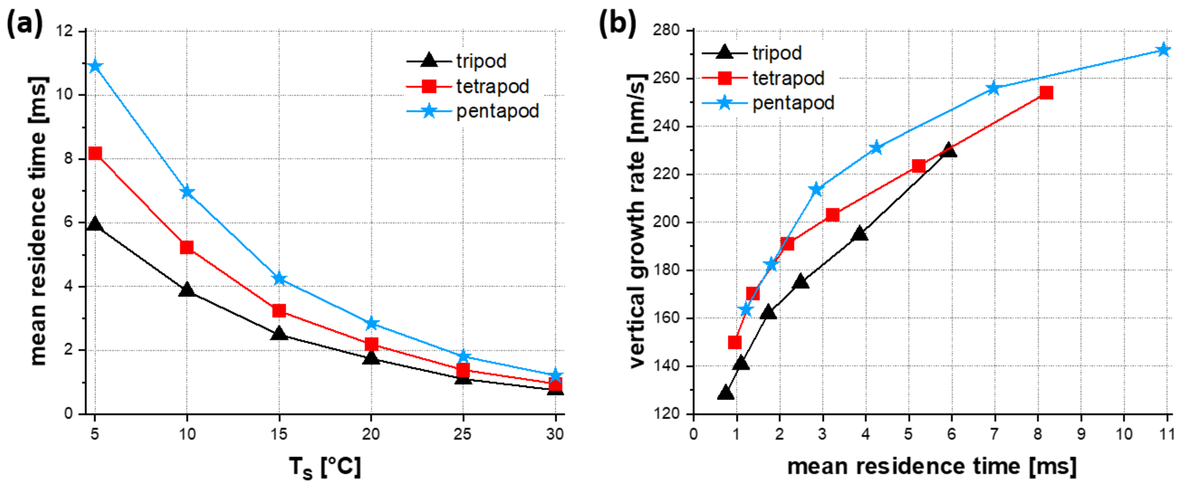

Figure S8. (a) mean residence time of precursor molecules at the pillar growth front. **(b)** experimental found vertical growth rate as a function of the mean residence time.

References

[1] E. Mutunga, R. Winkler, J. Sattelkow, P.D. Rack, H. Plank, J.D. Fowlkes, Impact of Electron-Beam Heating during 3D Nanoprinting, ACS Nano. 13 (2019) 5198–5213. https://doi.org/10.1021/acsnano.8b09341.

[2] R. Winkler, J.D. Fowlkes, P.D. Rack, G. Kothleitner, H. Plank, Shape Evolution and Growth Mechanisms of 3D-printed Nanowires, Addit. Manuf. (2021). In press. https://doi.org/10.1016/j.addma.2021.102076
